# Ectopic expression of *OsMADS45* activates the upstream genes *Hd3a* and *RFT1* at an early development stage causing early flowering in rice

**DOI:** 10.1186/1999-3110-54-12

**Published:** 2013-08-21

**Authors:** Jiun-Da Wang, Shuen-Fang Lo, Yan-Suan Li, Po-Ju Chen, Shih-Yun Lin, Teh-Yuan Ho, Jenq-Horng Lin, Liang-Jwu Chen

**Affiliations:** 1 Department of Life Sciences, National Chung Hsing University, Taichung 402, Taiwan; 2 Institute of Molecular Biology, National Chung Hsing University, Taichung 402, Taiwan; 3 Agricultural Biotechnology Center, National Chung Hsing University, Taichung 402, Taiwan; 4 Institute of Molecular Biology, Academia Sinica, Taipei 115, Taiwan; 5 Institute of Plant and Microbiology, Academia Sinica, Taipei 115, Taiwan

**Keywords:** Floral regulatory genes, *Hd1*, *Hd3a*, *OsMADS45*, *RFT1*, Rice

## Abstract

**Background:**

The rice gene, *OsMADS45*, which belongs to the MADS-box E class gene, participates in the regulation of floral development. Previous studies have revealed that ectopic expression of *OsMADS45* induces early flowering and influences reduced plant height under short-day (SD) conditions. However, the regulation mechanism of *OsMADS45* overexpression remains unknown. We introduce an *OsMADS45* overexpression construct *Ubi:OsMADS45* into TNG67 plants (an *Hd1* (*Heading date 1*) and *Ehd1* (*Early heading date 1*) defective rice cultivar grown in Taiwan), and we analyzed the expression patterns of various floral regulators to understand the regulation pathways affected by *OsMADS45* expression.

**Results:**

The transgenic rice exhibit a heading date approximately 40 days earlier than that observed in TNG67 plants, and transgenic rice display small plant size and low grain yield. *OsMADS45* overexpression did not alter the oscillating rhythm of the examined floral regulatory genes but advanced (by approximately 20 days) the up-regulate of two florigens, *Hd3a* (*Heading Date 3a*) and *RFT1* (*RICE FLOWERING LOCUS T1*) and suppressed the expression of *Hd1* at the juvenile stage. The expression levels of *OsMADS14* and *OsMADS18*, which are two well-known reproductive phase transition markers, were also increased at early developmental stages and are believed to be the major regulators responsible for early flowering in *OsMADS45*-overexpressing transgenic rice. *OsMADS45* overexpression did not influence other floral regulator genes upstream of *Hd1* and *Ehd1*, such as *OsGI (OsGIGANTEA)*, *Ehd2/Osld1/RID1* and *OsMADS50*.

**Conclusion:**

These results indicate that in transgenic rice, *OsMADS45* overexpressing ectopically activates the upstream genes *Hd3a* and *RFT1* at early development stage and up-regulates the expression of *OsMADS14* and *OsMADS18*, which induces early flowering.

**Electronic supplementary material:**

The online version of this article (doi:10.1186/1999-3110-54-12) contains supplementary material, which is available to authorized users.

## Background

The plant developmental transition from vegetative to reproductive growth is regulated by multiple genes and environmental factors, such as temperature and photoperiod (Koornneef et al. 1998; Wilczek et al. 2010). In rice, this transition influences the time of floral heading. The heading date is an important agronomic trait of cultivated rice for adaption to variable growth season and photoperiod changes and is also associated with biomass, plant size and grain productivity (Xue et al. 2008; Endo-Higashi and Izawa 2011). Rice are cultivated within a wide range of latitudes ranging from 55°N to 36°S; therefore, a wide range of heading varieties are required for efficient and sustainable agriculture practices in these various geographic regions (Khush 1997). In rice, the flowering time or heading date is primarily controlled by the two florigens, Hd3a and RFT1 (Kojima et al. 2002; Komiya et al. 2008). Hd3a, an ortholog of Arabidopsis FT, interacts with 14-3-3 proteins in the apical cells of shoots, thereby yielding a complex that translocates to the nucleus and binds to OsFD1, which is a rice transcription factor homologue of Arabidopsis FD, to induce floral initiation (Taoka et al. 2011; Tsuji et al. 2013). RFT1, another ortholog of Arabidopsis FT, is a major floral activator under long-day (LD) conditions. The defective expression of RFT1 increases the heading date to more than 200 days, while, RFT1 overexpression reduces the heading date (Komiya et al. 2008; Komiya et al. 2009). During flower development, Hd3a and RFT1 translocate from the leaf to the apical meristem, thereby activating the expression of downstream genes, such as *OsMADS14*, *OsMADS15* and *OsMADS18,* which regulate flower development (Tamaki et al. 2007; Komiya et al. 2008; Komiya et al. 2009; Kobayashi et al. 2012).

The regulation of *Hd3a* and *RFT1* transcriptions involving multiple flowering pathways under SD and LD conditions have been reported (Greenup et al. 2009). In the OsGI-Hd1-Hd3a pathway, OsGI, similar to the Arabidopsis flowering time gene GIGANTA (GI), acts as a positive regulator upstream of Hd1 under both LD and SD conditions (Hayama et al. 2003). Hd1, which is orthologous to Arabidopsis CONSTANS (CO), regulates the expression of *Hd3a*. Hd1 stimulates the expression of *Hd3a* under SD conditions but represses *Hd3a* expression under LD conditions (Hayama and Coupland 2004). In the Ehd2-OsMADS50-Ehd1-RFT1 flowering pathway, Ehd1 represents another important expression regulator of *Hd3a* and *RFT1*. Ehd1 not only up-regulates the expression of *Hd3a* under SD conditions but also activates the expression of *Hd3a* and *RFT1* under LD conditions (Doi et al. 2004; Komiya et al. 2009). *Ehd1* expression is regulated by multiple floral regulators, such as OsMADS50, OsMADS51, OsMADS56 and Ehd2/Osld1/RID1. Under SD conditions, the floral signal of OsGI is transmitted by OsMADS51, which is a MADS-box gene downstream of OsGI, to induce the expression of *Ehd1* (Kim et al. 2007). Under LD conditions, *Ehd1* is also up-regulated by OsMADS50 and Ehd2/Osld1/RID1, which is a Cys-2/His-2-type zinc finger transcription factor, while *Ehd1* expression is repressed by OsMADS56 (Matsubara et al. 2008; Park et al. 2008; Wu et al. 2008; Ryu et al. 2009). Thus, the expression of *Hd3a* and *RFT1* is regulated by multiple regulatory networks that govern flower initiation.

Rice MADS-box genes, which are required for floral organ differentiation and flowering time regulation, are grouped into the classes A, B, C, D and E based on the sequences homology and functional similarities to Arabidopsis homologues (Yamaguchi and Hirano 2006; Ciaffi et al. 2011). The MADS-box class A genes, *OsMADS 14*, *15* and *18*, are downstream in the flowering signaling pathway and are regulated by Hd3a and RFT1 under SD and LD conditions (Komiya et al. 2009; Kobayashi et al. 2012). Low expression levels of *OsMADS14*, *OsMADS15* and *OsMADS18* have been observed in the leaves at early developmental stages, while the expression of these genes increases when the plants reach the reproductive stage (Lee et al. 2004; Komiya et al. 2008; Kim et al. 2008). OsMADS14 and OsMADS15, which are homologous to Arabidopsis APETALA (AP1), which share a 72% amino acid sequence identity, interact with the E class protein, OsMADS1, during flower development regulation (Lim et al. 2000). *OsMADS14* overexpression significantly reduces flowering time and causes flower bud development as early as transgenic rice cultured in regeneration medium (Jeon et al. 2000a). An *OsMADS14* ortholog mutant, *maintained vegetative phase* (*mvp)*, displayed no transition from vegetative to reproductive phase in einkorn wheat (*Triticum monococcum*) (Shitsukawa et al. 2007; Tsuji et al. 2008). Additionally, the overexpression of *OsMADS18* revealed a short heading date in transgenic rice, while the heading date did not change in the knockout mutant (Fornara et al. 2004). For *OsMADS15*, a similar functional assay has not yet been reported. Therefore, the expression of *OsMADS14* and *OsMADS18* are closely associated with the reproductive phase transition. The expression analysis of these genes may elucidate the mechanism of the reproductive stage transition.

Rice *OsMADS45* (also known as *OsMADS7*), which belongs to class E, is specifically expressed in the stamen, pistil, and carpel, but not in the leaves and other vegetative tissues (Greco et al. 1997; Arora et al. 2007; Bai et al. 2008). E-functional genes are required for specifying organ identity of the inner three whorls of the floral organs in combination with A-, B-, C- and D-functional genes (Pelaz et al. 2000; Honma and Goto 2001). Many studies have indicated that OsMADS45 interacts with other MADS proteins to co-regulate the determination of floral organ identities (Seok et al. 2010). OsMADS13, which is a D class gene, interacts with OsMADS45 at the protein level to control ovule development during flower growth (Favaro et al. 2002; Dreni et al. 2007). OsMADS45 also associates with the B class proteins, OsMADS16 and OsMADS4, to form an OsMADS16-OsMADS4-OsMADS45 ternary complex and regulate the development of the lodicule and other floral organs (Moon et al. 1999a; Lopez-Dee et al. 1999; Seok et al. 2010). In addition to floral identity determination, the overexpression or deletion of E genes has been shown to alter the flowering time (Jeon et al. 2000a; Jeon et al. 2000b; Cui et al. 2010). For example, the *OsMADS45* and *OsMADS8* knockout plants displayed late flowering and a loss of floral identity (Cui et al. 2010), while the overexpression of *OsMADS45* resulted in early flowering and affected other agricultural characteristics in transgenic rice (Jeon et al. 2000a). Similarly, a T-DNA activation-tagged mutant that has *OsMADS45* activated and expressed in leaves and other tissues revealed early flowering and the same early flowering phenotype was observed by overexpressing *OsMADS45* in a Taiwan rice variety TNG67. TNG67 is known as a photoperiod insensitive cultivar containing defective *Hd1* and *Ehd1* sequences as those of T65 (Doi et al. 2004), and malfunction of these two genes *Hd1* and *Ehd1* delayed heading date up to 6.8 days and 17.4 days, respectively (Chen et al. 2010; Chien et al. 2011). However, the regulation mechanism causing early flowering in these *OsMADS45* overexpression transgenic rice remains unknown. Thus, we expressed the *OsMADS45* gene ectopically in TNG67 with the *hd1* and *ehd1* genetic background to analyze the early flowering enhancing mechanism and to demonstrate the probable effects of *OsMADS45* in regulating the flowering time when it was overexpressed. The overexpression of *OsMADS45* not only induced the early expression of *Hd3a* and *RFT1* but also reduced the expression of *Hd1* at early developmental stages. Based on these data, we propose a model that explains the mechanism by which the ectopic expression of *OsMADS45* causes early flowering in rice.

## Methods

### Plant material and growth conditions

The rice cultivar, *Oryza sativa* L. cv. TNG67, was analyzed in this study. The plants used for agronomic trait measurements were grown in an experimental field in Taichung, Taiwan (24° 4′ 40.2″ N; 120° 43′ 0.61″ E). The plants used for the study of diurnal gene oscillations were grown in a growth chamber under a 14 h light/10 h dark cycle condition using fluorescent white light tubes (400 to 700 nm, 100 μmol m^-2^ s^-1^) at 28°C.

### Measurement of photosynthesis rate

The fully expanded upper three leaves of 60 DAI (days after imbibition) plants were used to measure the photosynthetic rate. The rate of photosynthetic CO_2_ assimilation was measured with a portable Plu-LCi ultra compact photosynthesis system (ADC BioScientific Ltd., Hertfordshire, UK). The measurements were performed from 9 AM to 3 PM with an external constant light source (Ess Capsule G4, Royal Philips Electronics, Amsterdam, Dutch). During the measurement procedure, the leaf temperature was 37°C, the reference CO_2_ was 390 ppm, the relative air humidity was 60%, and the flow rate to the leaf chamber was 300 mL min^-1^.

### Plasmid construction and rice transformation

The full-length *OsMADS45* cDNA was cloned by PCR amplification with the primer pair, BamOsMADS45, which containing a*Bam* HI digesting site (Additional file 1: Table S1) and introduced into a pGEM-T easy vector (Promega, http://www.promega.com/). The *OsMADS45* cDNA was sub-cloned into a PLN vector containing the maize ubiquitin promoter (Ubi) and the *nos* terminator (Sun and Gubler 2004). The full-length construct (*Ubi*:*OsMADS45*) was fused with the pCAMBIA1301 vector, and the *Agrobacterium tumefaciens* strain, EHA105, was used for the rice transformations. Calli induced from seeds were co-cultured with *A. tumefaciens*, and the putative transgenic rice was regenerated from calli as described previously (Chen et al. 2002).

### Genomic DNA extraction and genes sequencing

Total genomic DNA extraction was performed as described previously (Lin et al. 2009). The *Hd1* and *Ehd1* gene fragments were amplified by PCR with Promega Taq DNA polymerase. Two primer pairs (Hd1-1 and Hd1-2) were used to sequence the *Hd1* gene. The primer pair Ehd1-1 was used to sequence the *Ehd1* gene (Additional file 1: Table S1).

### Southern blot analysis

Fifteen microgram of genomic DNA was digested with *Nco* I and separated on a 1% agarose gel. A *GUS* DNA fragment was amplified via PCR with the primer pair, GUS (Additional file 1: Table S1), and was used as probe. The GUS fragment was labeled with (α-P^32^) dCTP using the Rediprime™ II DNA Labeling System (GE, http://www.gelifesciences.com/). Southern blot analysis was performed as described previously (Ho et al. 2000).

### RNA isolation and semi-quantitative RT-PCR

Total cellular RNA was extracted using TRIzol reagent (Invitrogen, http://www.invitrogen.com/), and the residual DNA was digested with RQ1 RNase-Free DNase (Promega) to minimize the contamination. For semi-quantitative RT-PCR, first-strand cDNA was synthesized from 2 μg of total RNA using M-MLV reverse transcriptase (Promega) in a 25 μL reaction mixture containing 0.5 μg of dT18 primer and 20 units of recombinant RNase inhibitor (TaKaRa, http://www.takara-bio.com/) according to the manufacturer’s instructions. For the quantification of cDNA, rice *Actin1* (*Act1*; Lin et al. Os03g0718100; Lin et al. 2009) was used to normalize the expression levels. The gene-specific primer pairs are listed in Additional file 1: Table S1. The PCR reactions involved 5 min of denaturation at 95°C, followed by 95°C for 30 sec, 62°C (54°C for *Ehd1* and *Hd3a*) for 30 sec and 72°C for 45 sec. The PCR cycles used for each amplification are as follows: *Act1*, 28 cycles; *Ehd1*, 34 cycles; *Ehd2*, 34 cycles; *Hd1*, 34 cycles; *Hd3a*, 34 cycles; *OsGI*, 34 cycles; *OsMADS7*, 26 cycles; *OsMADS14*, 34 cycles; *OsMADS18*, 34 cycles; *OsMADS50*, 28 cycles and *RFT1*, 34 cycles.

## Results

### Ectopic expression of *OsMADS45* causes early flowering in the TNG67 rice variety

**Figure 1:**
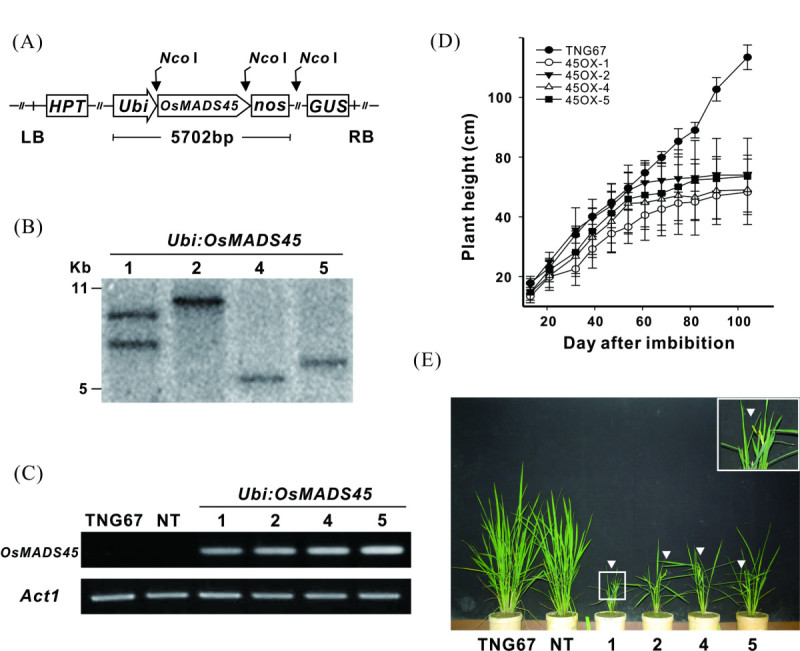
**The transformation vector and the molecular and phenotype analysis of the** ***Ubi:OsMADS45*** **transgenic rice. (A)** A schematic diagram of the transformation vector, *Ubi:OsMADS45*. Full-length *OsMADS45* cDNA driven by an ubiquitin (Ubi) promoter, and the nos terminator was inserted into the plasmid vector, pCAMBIA1301. **(B)** Southern blot analysis of four selected *Ubi:OsMADS45* transgenic plants. Total genomic DNA isolated from four transgenic lines (45OX1, 45OX2, 45OX4, and 45OX5) was digested with *Nco* I and hybridized with a GUS DNA probe. **(C)** RT-PCR analysis of *Ubi:OsMADS45* transgenic rice. Total RNA was isolated from 20 DAI leaves and analyzed using PCR primers specific for the *OsMADS45* gene. Actin expression was assessed as an internal control. **(D)** The plant heights of TNG67 rice and the four transgenic lines from 20 to 100 DAI. The heights of 12-16 plants for each line were measured, and the error bars indicate the SE of the mean at each time point. **(E)** Morphological characteristics of TNG67 rice, non-transgenic (NT) and transgenic lines at 74 DAI. Arrowheads indicate the flowering panicles. Inset: an enlargement from the square portion indicated in line 1. DAI, days after imbibition; GUS, beta-glucuronidase; and HPT, hygromycin phosphor transferase.

To analyze the early flowering enhancing mechanism of *OsMADS45* and to demonstrate the probable effects of *OsMADS45* in regulating the flowering time when it was overexpressed, we ectopically overexpressed *OsMADS45* in the rice variety, TNG67, by transforming the *Ubi*:*OsMADS45* construct containing the *OsMADS45* gene driven by the maize ubiquitin promoter (Figure 1A) into the plant. After the transformation, more than 10 transgenic lines displayed early flowering and stunted growth, and this early flowering phenotype associated with *OsMADS45* expression observed in the progenies of various transgenic lines was analyzed and verified for more than three generations. Four independent transgenic lines confirmed by Southern blot assay (Figure 1B) were selected for further investigation. RT-PCR analysis using RNA extracted from 20 DAI leaves revealed the overexpression of the *OsMADS45* gene in all four *Ubi*:*OsMADS45* transgenic lines (designated as 45OX1, 45OX2, 45OX4 and 45OX5), while no expression was detected in the wild-type (TNG67) and non-transgenic (NT) lines (Figure 1C). Although the RNA expression levels of *OsMADS45* for these four transgenic lines were similar, the plant heights varied slightly (Figure 1D and E and Table 1).

**Table 1:** **Agronomic traits of TNG67 and** ***Ubi:OsMADS45*** **transgenic rice plants**

Characteristics	TNG67^a^	45OX-1^a^	45OX-2^a^	45OX-4^a^	45OX-5^a^
Heading date ^b^	89 ± 3	51 ± 2	46 ± 3	48 ± 4	50 ± 4
Plant height ^c^ (cm)	93.4 ± 4.1	48.3 ± 6.4	54.0 ± 5.3	49.1 ± 11.6	53.6 ± 12.8
Panicle length (cm)	20.2 ± 2.3	11.3 ± 1.0	12.7 ± 2.2	11.6 ± 2.7	11.5 ± 2.0
Tiller number ^c^	23 ± 4	13 ± 3	14 ± 3	15 ± 4	16 ± 3
Fertility rate ^d^ (%)	95.8 ± 1.8	16.6 ± 9.2	45.2 ± 15.9	30.7 ± 22.3	37.6 ± 20.1
WTS ^e^ (g)	22 ± 0.8 (100%)	14.8 ± 2.0 (67%)	16.2 ± 1.3 (73%)	16.9 ± 0.8 (76%)	14.6 ± 0.6 (66%)
Number of spikelets per panicle	105 ± 15 (100%)	52 ± 22 (50%)	52 ± 12 (50%)	61 ± 15 (58%)	53 ± 17 (50%)
Grain yield ^f^ (g)	44.7 ± 7.7 (100%)	3.5 ± 2.0 (8%)	8.5 ± 3.2 (19%)	7.5 ± 6.7 (17%)	8.1 ± 4.5 (18%)
Shoot dry weight ^g^ (g)	46.9 ± 8.6 (100%)	10.8 ± 3.6 (23%)	17.7 ± 4.4 (38%)	14.9 ± 8.8 (32%)	16.3 ± 8.3 (35%)
PR ^h^ (μmole m^-2^ s^-1^)	10.5 ± 1.1	12.7 ± 2.4	13.2 ± 2.2	11.4 ± 2.3	10.4 ± 2.4

^a^ The plant number of TNG67 was 27, 45OX-1 was 15, 45OX-2 was 13, 45OX-4 was 20, and 45OX-5 was 20.

^b^ Heading date was the date when the panicles protruding from the flag leaf sheath.

^c^ Plant height and total tiller number were measured on 104 DAI.

^d^ Fertility rate was the percentage of spikelets that set fertile seeds.

^e^ Weight of one thousand seeds (WTS) was the weight counted with 50 seeds and multiplied by 20.

^f^ Grain yield was the total weight of grains produced from one plant.

^g^ Shoot dry weight was the weight of the up-ground part of rice without the grains.

^h^ Photosynthetic rate (PR) was measured at noon on 68 DAI.

The progenies of these four transgenic lines were grown in an isolated field for two crop seasons, and their agronomic traits, including heading date, plant height, panicle length, tiller number, fertility rate, grain size, number of spikelets per panicle, grain yield, shoot dry weight and photosynthetic rate, were measured and collected (Figure 1D and Table 1). The heading dates of the transgenic lines ranged from 46 to 51 DAI, which is approximately 40 days earlier than that of TNG67 plants (heading at 89 DAI) (Table 1). The difference in plant height between TNG67 and the transgenic lines was not significant until approximately 50 DAI, when the transgenic lines started heading (Figure 1D). The plant height of transgenic lines was retarded after 50 DAI, and the plants essentially stopped growing after 60 DAI (Figure 1D). The final plant height of the transgenic lines (48-54 cm) was approximately 40 cm shorter than that of the TNG67 plants (93 cm). The transgenic plants measured approximately 50% of the TNG67 panicle length and reduced tiller number (13-15 vs. 23 tillers) and lower fertility rates (17-45% vs. 96%) (Table 1). Additionally, we found that the transgenic plants per grain weight measured 66-76% of that of the TNG67 plants, the number of spikelets per panicle for the transgenic plants was 50-58% of that observed with the TNG67 plants, and the grain yield for the transgenic plants was 8-19% of that observed with the TNG67 plants (Table 1). In terms of biomass production, the shoot dry weight of the transgenic plants was approximately 23-38% of that measured with the TNG67 plants; however, no difference in the photosynthetic rate was observed between the TNG67 plants and the transgenic lines.

### Ectopic expression of *OsMADS45* does not alter the oscillation rhythm of flowering time regulators

**Figure 2:**
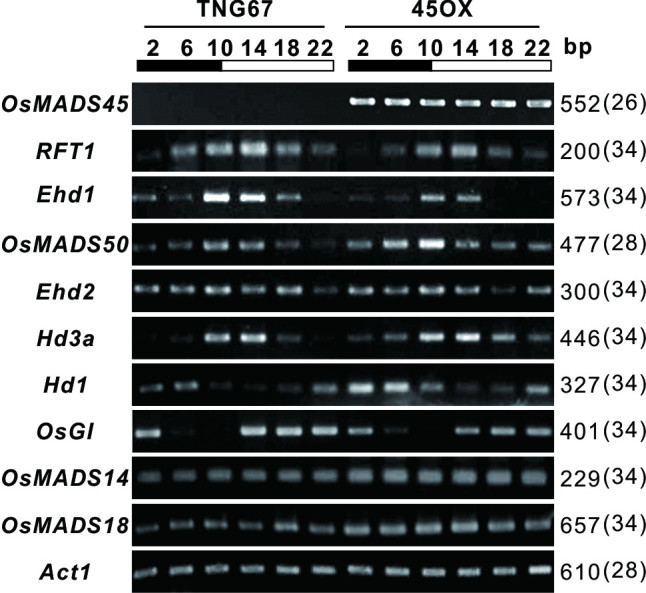
**The diurnal expression patterns of the floral regulatory genes of the Ehd2-OsMADS50-Ehd1-RFT1 and OsGI-Hd1-Hd3a flowering pathways.** Leaf RNA samples from 60 DAI of TNG67 rice and *Ubi:OsMADS45* transgenic rice (45OX) grown under 14 h light/10 h dark cycle condition at 4-hour intervals were collected and analyzed via RT-PCR using gene-specific primers (Additional file 1: Table S1). The actin gene was assessed as an internal control. The black bars indicate the dark period and the white bars indicate the light period.

Flowering in many plants (including rice) is dependent on the day length. LD and SD photoperiod-sensitive rice initiate flowering dependent on a critical threshold associated with the proportion of diurnal hours. The light signals intercepted by photoreceptors and the length of photoperiod determined by the circadian clock regulators are integrated and lead to either the induction or suppression of flowering (Izawa, 2007). The floral regulators expressed in OsGI-Hd1-Hd3a and Ehd2-OsMADS50-Ehd1-RFT1 flowering pathways are involved in the oscillation rhythm and controlled the flowering time (Ryu et al. 2009; Komiya et al. 2009). To study the oscillatory patterns of these floral regulators in 45OX-5 transgenic rice, leaf RNA samples were analyzed at each 4-hour interval under 14 h light/10 h dark cycle condition. The results showed that the diurnal oscillations of the floral regulators, including the light-induced *Hd3a, RFT1* and *Ehd1* regulators and the dark-induced *Hd1* regulators, remained constant in 45OX transgenic lines (Figure 2). This result suggests that ectopic expression of *OsMADS45* does not alter the diurnal oscillations of the examined floral regulators in 45OX transgenic rice on a TNG67 genetic background.

### Expression analysis of various floral regulatory genes in 45OX transgenic rice

**Figure 3:**
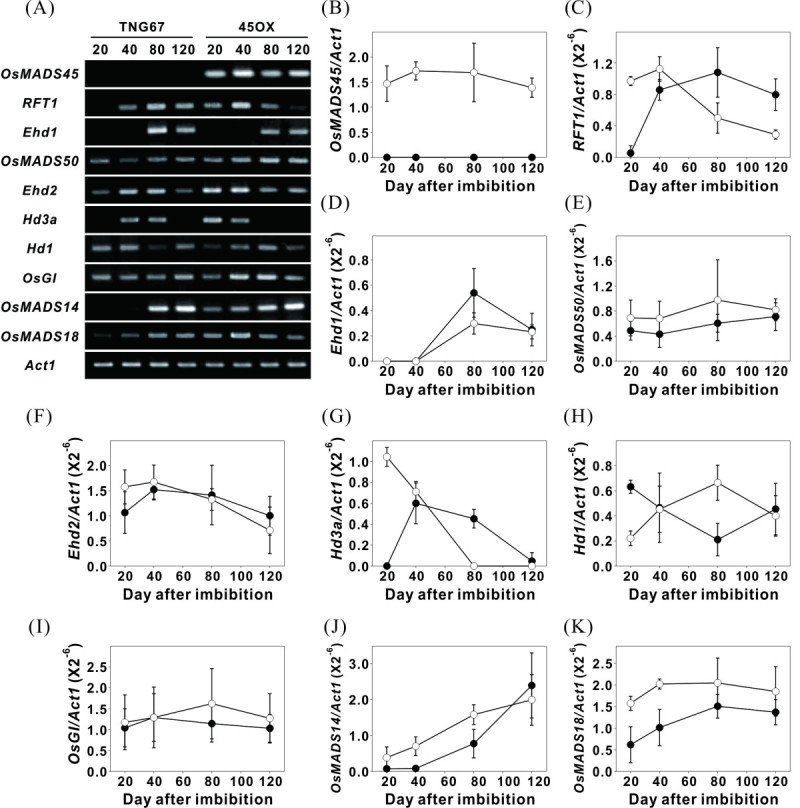
**Expression analysis of the floral regulatory genes** ***Ehd2, OsMADS50, Ehd1, RFT1, OsGI, Hd1, Hd3a*** **,** ***OsMADS14*** **and** ***OsMADS18*** **in TNG67 and** ***Ubi:OsMADS45*** **transgenic rice at various growth stages. (A)** Representative RT-PCR analysis of the floral regulatory genes expression in TNG67 and *Ubi:OsMADS45* transgenic plants (45OX) at various growth stages is shown. Leaf RNA was isolated from the plant samples of TNG67 and *Ubi:OsMADS45* transgenic rice (45OX) approximately 2 to 4 hours after or before (for *Hd1* gene analysis) dawn at 20, 40, 80 and 120 DAI; the RNA was analyzed via RT-PCR using gene-specific primers (Additional file 1: Table S1). *Act1* gene was used as an internal control. **(B-K)** Semi-quantitative RT-PCR data of *OsMADS45* **(B)**, *RFT1* **(C)**, *Ehd1* **(D)**, *OsMADS50* **(E)**, *Ehd2* **(F)**, *Hd3a* **(G)**, *Hd1* **(H)**, *OsGI* **(I)**, *OsMADS14* **(J)**, and *OsMADS18* **(K)** in TNG67 (black circle) and 45OX (white circle) plants are shown. The Y axes indicate the relative transcript levels of each gene. The transcript levels were quantified and normalized against actin RNA. The error bars indicate the SE for three replicate experiments.

In rice, two independent photoperiod pathways, involving the floral regulators, Hd1 and Ehd1, control flowering time via the regulation of Hd3a (Komiya et al. 2009). *Hd1* expression is predominantly regulated by the circadian clock through OsGI signaling (Hayama et al. 2003), which regulates *Ehd1* expression under SD conditions (Kim et al. 2007). Hd1, Ehd1 and Hd3a, are known to play a key role in photoperiod-controlled heading. Additionally, the OsMADS50-Ehd1-RFT1 pathway is involved in floral activation under LD conditions (Komiya et al. 2009). To understand the mechanism by which these floral regulators promote early flowering of the 45OX transgenic rice, we isolated RNA from the leaves at various growth stages, including 20, 40, 80, and 120 DAI, and compared the expression levels with those of the wild-type TNG67. The results showed the expression patterns of the *RFT1*, *Hd3a*, *Hd1*, *OsMADS14* and *OsMADS18* genes in the 45OX transgenic rice varied from those observed with the TNG67 rice (Figure 3A), thereby suggesting that these genes may be involved in the process of early flowering in the 45OX transgenic rice. The expression levels of the floral regulatory genes observed for the two flowering pathways are detailed in the following sections.

### The expression of *RFT1* is activated at early developmental stages in 45OX transgenic rice

The expression of the *Ehd2*, *MADS50*, *Ehd1*, and *RFT1* genes of the Ehd2-OsMADS50-Ehd1-RFT1 flowering pathway were analyzed. *RFT1* is a major floral activator under LD conditions, and its defective expression increases the heading date to above 200 days (Komiya et al. 2009), while, *RFT1* overexpression reduces the heading date (Komiya et al. 2008). In the present study, the expression levels of *RFT1* in 45OX transgenic rice were significantly higher than that observed in the TNG67 rice on 20 DAI. On 40 DAI, the *RFT1* transcript levels observed in the TNG67 rice increased and reached the same levels of those observed in the 45OX transgenic rice. However, after 40 DAI, *RFT1* expression levels decreased in the 45OX transgenic rice and increased in the TNG67 rice (Figure 3A and C).

*Ehd1*, a B-type response regulator, is an upstream positive regulator of *RFT1* under LD conditions (Komiya et al. 2009), which showed no expression on 20 and 40 DAI and increased expression after 80 DAI (Figure 3A and D). Its expression pattern in 45OX transgenic rice was similar to that observed in the TNG67 plants (Figure 3A and D). *OsMADS50*, a MICK-type MADS-box gene, is an epistatic active regulator of *Ehd1* that was expressed throughout all development stages and displayed the same expression pattern in 45OX transgenic rice and the TNG67 plants (Figure 3A and E). *Ehd2*/*Osld1*/*RID1*, a zinc finger transcription factor orthologous with maize *Indeterminate1* (*ID1*), is another upstream positive regulator of *Ehd1* under LD conditions, which was expressed in all stages and failed to show different expression patterns between the 45OX transgenic rice and TNG67 rice (Figure 3A and F).

These results indicate that the overexpression of *OsMADS45* activates the expression of *RFT1* at early development stages, but fails to affect the expression of the upstream genes. As the heading of 45OX transgenic rice and TNG67 plants were detected soon after the high accumulation of the *RFT1* transcript, we hypothesized that the activation and accumulation of *RFT1* during the early development stages induces early flowering in 45OX transgenic rice.

### The up-regulation of *Hd3a* and down-regulation of *Hd1* at early development stages were observed in 45OX transgenic rice

The expression of the *OsGI, Hd1* and *Hd3a* genes of the OsGI-Hd1-Hd3a flowering pathway were analyzed. *Hd3a*, which is a rice florigen, has been shown to bind several proteins to induce flowering (Kojima et al. 2002) and was significantly expressed in the 45OX transgenic rice on 20 DAI, while the expression was decreased slightly on 40 DAI and then vanished after 80 DAI (Figure 3A and G). In contrast, *Hd3a* expression was not detected in the TNG67 plants on 20 DAI, showed increased expression from 40 to 80 DAI, and then disappeared on 120 DAI after flowering.

*Hd1*, another floral regulator orthologous with *Arabidopsis CO*, is an upstream repressor of *Hd3a* under LD conditions. *Hd1* mRNA was approximately three-fold lower in the 45OX transgenic rice compared with the TNG67 rice on 20 DAI (Figure 3A and H). Nonetheless, no difference was observed on 40 DAI between the 45OX transgenic and TNG67 rice, the expression of *Hd1* increased slightly on 80 DAI and then decreased in the 45OX transgenic rice, while decreased after 40 DAI and on 80 DAI for in the TNG67 rice (Figure 3A and H).

*OsGI*, a rice ortholog of *Arabidopsis GI*, is a positive regulator upstream of *Hd1* that was expressed at all stages and failed to reveal different expression patterns between 45OX transgenic and TNG67 rice (Figure 3A and I). Our results indicate that *OsMADS45* overexpression potentiates its effects on the OsGI-Hd1-Hd3a flowering signaling pathway downstream of *OsGI* and upstream of the *Hd1* and *Hd3a* genes. The observations also reveal that the up-regulation of *Hd3a* at early development stages may play a role in the induction of early flowering in 45OX transgenic rice.

### The expression of *OsMADS14* and *OsMADS18* is up-regulated in 45OX transgenic rice

To gain insight into the mechanism underlying the increase in RFT1 in 45OX transgenic rice, we assessed the gene expression levels of *OsMADS14* and *OsMADS18*. *OsMADS14* and *OsMADS18*, which are two Arabidopsis AP1-like MADS-box class A genes, are expressed in the leaves and inflorescences. Expressions of *OsMADS14* and *OsMADS18* was detectable during the vegetative stages and surged when the plants reach the reproductive stages (Lee et al. 2004; Komiya et al. 2008; Kim et al. 2008). These genes are recognized as the most downstream genes regulated by *Hd3a* and *RFT1* under SD and LD conditions in both flowering-signaling pathways (Komiya et al. 2009). Although the apex-expressed but not the leaf-expressed *OsMADS14* and *OsMADS18* may directly participate in floral regulation, due to positive correlation between the leaf-expressed and apex- or floral-expressed *OsMADS14/18* has been confirmed (Komiya et al. 2009; Sato et al. 2011), the leaf-expressed *OsMADS14/18* were used in this study. Moreover, many previous studies used leaf-expressed *OsMADS14/18* to analyze the reproductive phase transition and floral regulation as well (Lee et al. 2004; Doi et al. 2004; Kim et al. 2007; Komiya et al. 2008; Kim et al. 2008; Tanaka et al. 2011).

The expression of *OsMADS14* was increased gradually from 20 DAI until 120 DAI in both the 45OX transgenic and TNG67 rice (Figure 3A and J). In the 45OX transgenic rice, similar expression levels were observed throughout all stages, while *OsMADS18* expression increased slowly from 20 to 120 DAI in TNG67 (Figure 3A and K). When comparing the expression levels of *OsMADS14* and *OsMADS18* in the 45OX transgenic and TNG 67 rice, we found that both genes were expressed at relatively higher levels in 45OX transgenic rice compared with the TNG67 rice throughout all stages with increasingly significant differences observed during the early stages (Figure 3A, J, and K). As no *OsMADS14* mRNA and relatively low levels of *OsMADS18* were detected at the early development stages (20 to 40 DAI) in the wild-type TNG67 rice, the increased expression of *OsMADS14* and *OsMADS18* in 45OX transgenic rice may play a critical role in promoting the early transition from the vegetative stage to the reproductive stage, thereby resulting in early flowering.

### Genes *Hd1* and *Ehd1* are defective and an alternative spliced *Hd1* mRNA was identified in TNG67 rice

**Figure 4:**
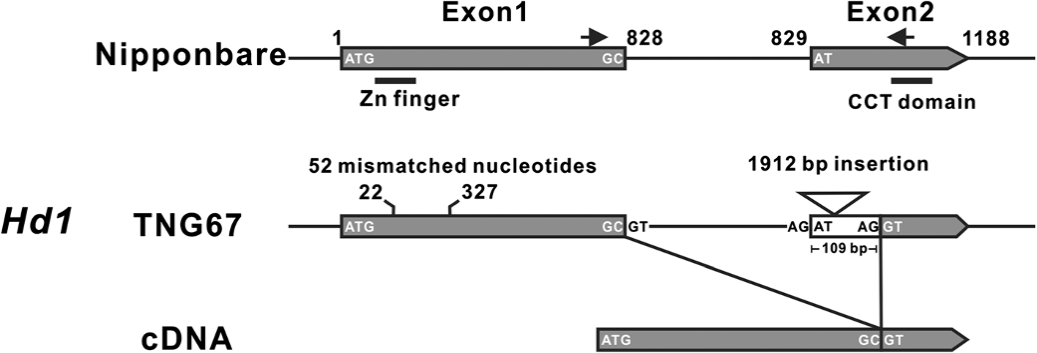
**A schematic diagram showing the sequence variations and cDNA of** ***Hd1*** **of TNG67 rice compare with Nipponbare plants.** In TNG67 rice, a total of 52 in/del and mismatched bases scattered between 22^nd^ and 327^th^ bases near the *Hd1* zinc finger domain in exon 1 and a 1912 bp insertion adjacent to the CCT domain in exon 2 are shown (see detail in Additional file 2: Figure S1). The *Hd1* cDNA of TNG67 with a potential alternative splice site located in exon 2 that causes the removal of a 109-bp of exon 2 and the 1912 bp insertion fragment is shown.

The *Hd1* gene is highly variable in the cultivated rice *Oryza sativa*. The various alleles of *Hd1* identified in the rice are believed to be selected by humans to adapt to the variable growing photoperiods (Izawa 2007; Takahashi and Shimamoto 2011). *Hd1* and *Ehd1* are key flowering time regulatory genes in rice; however, both genes have been identified to be non-functional in a Taiwan rice cultivar, Taichung 65 (T65), which shows little or no sensitivity to photoperiods (Doi et al. 2004). TNG67 is a hybrid cultivar descended from T65 plants and had decreased sensitivity to photoperiods. To verify *Hd1* and *Ehd1* gene integrity, both genes were sequenced in TNG67 rice. A total of 52 insertions or deletions and mismatched bases scattered between the 22^nd^ and 327^th^ bases near the *Hd1* zinc finger domain in exon 1 and a 1912-bp insertion adjacent to the CCT domain in exon 2 were identified (Figure 4 and Additional file 2: Figure S1). The same substitution (a functional glycine replaced by a non-functional arginine) in the 219^th^ amino acid of Ehd1 found in T65 rice was also detected (Additional file 3: Figure S2). Although sequence variations in the *Hd1* and *Ehd1* genes were detected, the corresponding mRNAs were still regulated and expressed in TNG67 rice (Figures 2 and 3). We also found that the cDNA sequence of the *Hd1* gene is 109 nucleotides shorter than that found in Nipponbare (Figure 4 and Additional file 2: Figure S1). Based on this cDNA sequence data obtained from the analysis of TNG67 rice, we concluded that this *Hd1* transcript was generated by an alternative splice site (another AG inside the white box) located in exon 2 as indicated in Figure 4. These results suggest that the lack of function of *Hd1* and *Ehd1* in TNG67 rice (Chen et al. 2010; Chien et al. 2011) does not resulted from inhibited gene expressions but instead was caused either by alternative splicing and a DNA insertion into the *Hd1* gene or by an amino acid substitution in the *Ehd1* gene.

## Discussion

The expression of the *OsMADS45* gene is not only involved in the determinations of floral organ identity (Favaro et al. 2002; Dreni et al. 2007; Seok et al. 2010) but also involved in the regulation of flower initiation that affects the heading date of rice (Jeon et al. 2000a; Cui et al. 2010). The heading date of *OsMADS45* RNAi knockout mutants was delayed by approximately 2 weeks (Cui et al. 2010). In contrast, the heading date of the *OsMADS45* overexpressing transgenic rice instead of being delayed was reduced from the 50 days observed in the wild-type Nakdong rice to 41-48 days in a controlled growth room with 10 h of light per day (Jeon et al. 2000a). For further comparison, the heading date observed in our 45OX transgenic rice was approximately 40 days (50 vs. 90 days) earlier than that of the wild-type rice (TNG67) under field conditions of 11.5-13.5 h of light per day. The discrepancy in the shortening the heading date between the Jeon et al. study and our study may be due to the different rice cultivars, Nakdong and TNG67, with different *Hd1* and *Ehd1* genetic background (Figure 4, Additional file 2: Figure S1 and Additional file 3: Figure S2).

The *Hd1* and *Ehd1* genes are involved in photoperiodic regulation during floral initiation (Doi et al. 2004; Izawa 2007; Endo-Higashi and Izawa 2011). As in T65 (Doi et al. 2004) and TK8 (Lin et al. 2011) cultivars, both the *Hd1* and *Ehd1* genes are defective in TNG67 (Figure 4, Additional file 2: Figure S1 and Additional file 3: Figure S2). However, the RNA expressions of the defective *Hd1* and *Ehd1* genes can be detected in TNG67 and 45OX transgenic rice (Figure 3); this finding deviates from the results of the T65 study that showed no detection of the *Hd1* RNA (Doi et al. 2004). While the transcript levels of *Hd1* (a *CO-like* gene) was altered in the 45OX transgenic rice, the expression of the *Ehd1* gene did not vary from that in the TNG67 (Figure 3A, D and H). In the Nakdong rice, both the *Hd1* and *Ehd1* genes were functionally expressed (Kim et al. 2008) and displayed photoperiod sensitivity (Jeon et al. 2000a). Therefore, we hypothesize that the varying effects in the shortening of the heading dates between the 45OX transgenic TNG67 and Nakdong rice may be a result of the regulation and expression of the *Hd1* gene. Our results showed that the *Hd1* gene was down-regulated during the juvenile stage in 45OX transgenic plants (Figure 3H), and when this phenomenon was observed in the Nakdong rice, which expressed a functional *Hd1* gene, would reduce the expression of *Hd3a* under SD conditions and abate the early flowering effect in transgenic Nakdong rice (Jeon et al. 2000a). However, additional analysis with our construct to transform Nakdong rice and grown in the same photoperiodic conditions would be required to address this discrepancy.

The floral time regulators in both the Ehd2-OsMADS50-Ehd1-RFT1 LD activation pathway and the OsGI-Hd1-Hd3a LD suppression pathway exhibit diurnal oscillation patterns (Matsubara et al. 2008; Ryu et al. 2009). We assessed the daily expression levels of these genes at each 4-hour interval, and the results showed that the expression patterns of the floral regulatory genes, such as *RFT1, Ehd1, OsMADS50, Ehd2, Hd3a, Hd1* and *OsG1* did not vary between the 45OX transgenic rice and the host plant TNG67 (Figure 2), thereby indicating that the ectopic expression of *OsMADS45* does not alter the diurnal oscillations of these genes. These observations suggest that the diurnal oscillations of these genes are not involved in reducing the flowering time in 45OX transgenic rice, and the normal diurnal oscillations of *RFT1* and *Hd3a* observed in the *hd1/ehd1* null cultivar, TNG67, imply that there are additional signal pathways other than those involving *Hd1* and *Ehd1* involved in the day/night rhythm of the *RFT1* and *Hd3a* genes*.*

Although the diurnal oscillations of the examined floral time regulators were not altered, the RNA expression levels of *RFT1* and *Hd3a,* which are two rice orthologs of the Arabidopsis *FT-* like gene, were up-regulated at a very early growth stages (20 DAI) in the 45OX transgenic rice (Figure 3). The up-regulation of the *FT* gene during different growth stages has been observed in transgenic Arabidopsis ectopically expressing MADS-box genes. For example, ectopic expression of orchid *MADS1* in Arabidopsis enhances the expression of *FT* and results in early flowering (Hsu et al. 2003), and the overexpression of *AGAMOUS*-*LIKE6* (*AGL6*, also a MADS-box gene) causes the precocious flowering phenotype by enhancing the expression of *FT* and the downstream gene *AP1* (Koo et al. 2010). Similarly, the expression of *RFT1* and *Hd3a* were up-regulated in the 45OX transgenic rice (Figure 3), thereby suggesting that the up-regulation of the *RFT1* and *Hd3a* genes at a very early growth stage (20 DAI) may contribute to the early flowering phenomenon observed in the 45OX transgenic rice.

*OsMADS14* and *OsMADS18* are downstream of *Hd3a* and *RFT1* and regulate the identity of floral meristem development (Ciaffi et al. 2011; Kobayashi et al. 2012). In the floral tissue, OsMADS45 directly interacts with OsMADS6 and OsMADS18; OsMADS6 may further interact with OsMADS14 and OsMADS18 (Moon et al. 1999b) and OsMADS6 and OsMADS18 may form a ternary complex with the histone fold protein, OsNF-YB1 (Masiero et al. 2002). These interactions suggest that OsMADS45, OsMADS6, OsMADS14, and OsMADS18 co-regulate floral development. A number of studies indicated that the expression of the *OsMADS14/15/18* were increased during the transition from the vegetative phase to the reproductive phase, thus, *OsMADS14/15/18* have been considered as reproductive phase transition markers (Lee et al. 2004; Komiya et al. 2008; Kim et al. 2008; Gao et al. 2013). A recent study revealed that a null mutant of *OsMADS6* (*Osmads6-5)* would down-regulate the expression of *OsMADS7/45*, but did not affect the expression of *OsMADS14/15/18*, therefore no flowering time change was observed (Duan et al. 2012). The same report showed over-expression of *OsMADS6* resulted in over production of various flower organs, but has little influence on vegetative traits or the heading date (Duan et al. 2012). However, in the present study, over-expression of *OsMADS45* resulted in an increased expression of *OsMADS14* and *OsMADS18* at early stages, approximately 20 to 40 days ahead of that of TNG67, and shortened the heading date and suppressed vegetative growth (Figure 3A, J and K). In contrast to 45OX transgenic rice that increased the expression of *OsMADS14/18* and *Hd3a*/*RFT1* and caused early flowering, a study using *MADS14*/*15*/*18* i-*pap2-1* quadruple knockdown mutant reduced the expression of *Hd3a*/*RFT1* and resulted in delayed flowering (Kobayashi et al. 2012). Accordingly, they proposed that *PAP2* and *AP1*-like genes, such as *MADS14*/*15*/*18,* function upstream of *Hd3a* and *RFT1* in leaves (Kobayashi et al. 2012). In summary, these observations suggested that 1) although OsMADS45 and OsMADS6 interact with OsMADS14 and OsMADS18 in regulating flower development, their effect on flowering time were different in overexpressing plants, 2) the downstream genes, such as *OsMADS14* and *OsMADS45* would regulate the expression of upstream genes (*Hd3a/RFT1*) in either knockdown or over-expression transgenic rice, and 3) the early expression of the *OsMADS14* and *OsMADS18* in 45OX transgenic rice shortened the vegetative growth period which resulted in a flowering transition much earlier than that observed in TNG67 rice.

**Figure 5:**
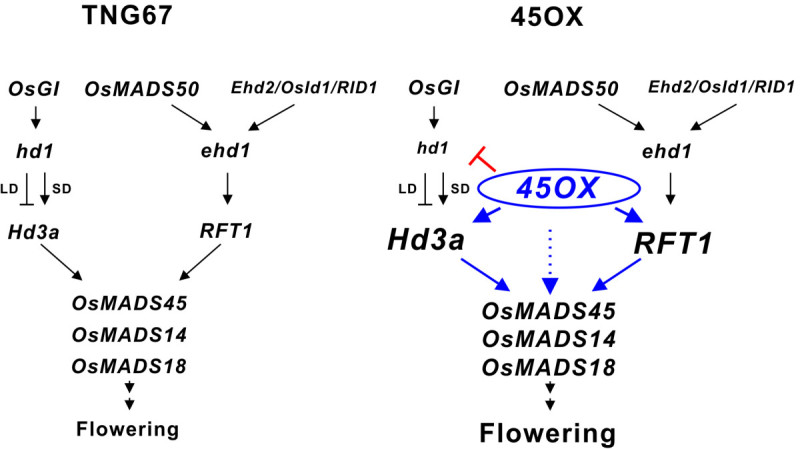
**A model of the early flowering signal pathway in 45OX transgenic rice.** The ectopic expression of *OsMADS45* activates the upstream genes *Hd3a* and *RFT1* at early developmental stages and upregulates the expression of *OsMADS14* and *OsMADS18* either via the upregulated of *Hd3a* and *RFT1* or direct induction via the constitutive expression of *OsMADS45*. The expression of *OsMADS45* represses the expression of *Hd1* at early developmental stages. The increased expression of the *OsMADS14* and *OsMADS18* genes caused early flowering in 45OX transgenic rice. However, the ectopic expression of *OsMADS45* does not alter the expression of *OsMADS50*, *Ehd1*, *Ehd2/OsId1/RID1* and *OsGI*.

It is interesting to point out that the expression levels of *RFT1* and *Hd3a* were dramatically decreased after heading at 50 DAI in 45OX transgenic rice or after heading at 90 DAI in TNG67 (Table 1; Figure 3C, G), and the expression of *RFT1* and *Hd3a* were not well correlated with the constitutively expression of *OsMADS45* in 45OX transgenic rice after heading (Figure 3B, C, G). The increased expression of *RFT1* and *Hd3a* at a higher level before heading and then at a significantly reduced level after heading were observed (Matsubara et al. 2008; Komiya et al. 2008; Kim et al. 2008; Ryu et al. 2009). These observations suggested that both the expression of *RFT1* and *Hd3a* were developmentally regulated by factors that could override the overexpression effect of *OsMADS45* after heading in 45OX transgenic rice*.* Although *OsMADS14* and *OsMADS18* are downstream of *Hd3a* and *RFT1,* the expression of *OsMADS14* and *OsMADS18* were not positively correlated with the expression of *Hd3a* and *RFT1* after heading (Kim et al. 2008). While the expression levels of *OsMADS14* and *OsMADS18* increased throughout the entire growth season, the expression of *Hd3a* and *RFT1* was reduced after heading and this pattern was also observed in TNG67 and in 45OX transgenic rice. This observation suggested that the increased expression of *OsMADS14* and *OsMADS18* after heading was controlled by factors in addition to *Hd3a* and *RFT1*. Based on the understanding of these flower development regulation pathways, we hypothesize that the early increased expression of the *OsMADS14* and *OsMADS18* genes in the 45OX transgenic rice may be caused by either the up-regulation of *Hd3a* and *RFT1* or directly induced by the constitutive expression of *OsMADS45* (Figure 5).

In the present study, we over-expressed the *OsMADS45* gene in TNG67 rice and showed that not only was the heading date reduced but also revealed that many other characteristics, such as plant height, shoot dry weight and the number of spikelets per panicle, were dramatically suppressed (Figure 1E and Table 1). A previous study, which involved the introducing of functional *Hd1* and/or *Ehd1* genes into T65 rice under different photoperiodic conditions, revealed that the combination of *Hd1* and *Ehd1* expression reduced the number of primary branches in a panicle, thereby resulting in smaller spiketlet numbers per panicle independent of the control of flowering time (Endo-Higashi and Izawa 2011). However, as observed in the T65 rice, TNG67 rice are defective in both the *Hd1* and *Ehd1* genes (Figure 4 and Additional file 3: Figure S2); therefore, the reduction in spikelet numbers per panicle and other characteristics observed in our 45OX transgenic rice could not have been affected by the function of *Hd1* and *Ehd1*. Additionally, no reduction in the photosynthetic rate was observed in 45OX transgenic rice (Table 1); this finding suggests that overexpression of *OsMADS45* does not affect the photosynthetic system and the reduction in shoot dry weight in 45OX transgenic rice was not caused by a reduction in photosynthesis. Furthermore, plant height and grain productivity associated with the flowering time regulators, *Ghd7* and *Ghd8*, have been previously reported (Xue et al. 2008; Yan et al. 2010). The report has shown that the enhanced expression of *Ghd7* under LD conditions could delay heading and increase both plant height and panicle size (Xue et al. 2008). Similarly, *Ghd8* plays a pleiotropic role in regulating grain productivity and heading date depending on its genetic background, which reveals a positive correlation between flowering time and grain productivity (Yan et al. 2010). Accordingly, we propose that many of the agronomic traits, such as shorter plant height, shorter panicle length, less tiller, less shoot dry weight and less grain yield in this 45OX transgenic rice might be the result of the effects of early flowering that reduced the vegetative growth periods from 89 days in TNG67 rice to 50 days.

## Conclusions

Based on our results, we propose a model to explain the signaling cascade in the early flowering phenotype of 45OX transgenic rice (Figure 5). The ectopic expression of *OsMADS45* activates the upstream genes, *RFT1* and *Hd3a*, at early development stages and up-regulates the expression of *OsMADS14* and *OsMADS18* via either the up-regulation of *Hd3a* and *RFT1* or direct induction via the constitutive expression of *OsMADS45,* thereby promoting early flowering. Additionally, the regulation and function of the various *Hd1* alleles may be involved in the different shortened effects on the heading dates observed between the 45OX transgenic TNG67 rice and Nakdong rice under the SD condition. In summary, our study illustrates the overexpression function of *OsMADS45* in initiating a possible bottom-up activation of the MADS-box genes signaling pathway that enhances early flower initiation, and the study using TNG67 rice may also represent another useful approach to study the flowering mechanism of other floral regulators to bypass the complex photoperiodic responses that are regulated by the *Hd1* and *Ehd1* signaling pathways.

## Electronic supplementary material

Additional file 1: Table S1: The primer pairs used in this study. (DOCX 18 KB)

Additional file 2: Figure S1: The genomic DNA sequence of the *Hd1* gene of TNG67 rice. Two *Hd1* exons are marked with boxes, and the intron junctions GT… AG are underlined and marked red. A total of 52 in/del and mismatched nucleotides in exon 1 (black boxed) compare with Nipponbare rice are marked with black (different bases) or grey shadings (additional bases in TNG67) or a dashed line to indicate the deleted bases. The red bases of exon 1 represent the zinc finger domain. A 1912-bp insertion is shaded grey within exon2 (red box), and the green bases represent the CCT domain. The proposed alternative splice site in exon 2 is underlined, in bold text and marked in orange. (TIF 18 MB)

Additional file 3: Figure S2: A schematic diagram showing the location of sequence variation and the detailed cDNA sequences of *Ehd1* of TNG67 rice. (A) The functional glycine (G) in Nipponbare rice replaced by a non-functional arginine (R) in the 219^th^ amino acid of Ehd1 as observed with T65 (Doi et al. 2004) and TK8 plants (Lin et al. 2011) is shown. (B) The cDNA sequences of *Ehd1* of TNG67 rice with the replaced DNA (underlined) and amino acid sequences (G to R). Five exons are marked with different colors, and the GARP domain is highlighted in yellow. (TIF 14 MB)

Below are the links to the authors’ original submitted files for images.

Authors’ original file for figure 1

Authors’ original file for figure 2

Authors’ original file for figure 3

Authors’ original file for figure 4

Authors’ original file for figure 5

Authors’ original file for figure 6

Authors’ original file for figure 7
