# Supplementary material for: Ectopic expression of OsMADS45 activates the upstream genes Hd3a and RFT1 at an early development stage causing early flowering in rice
Source: Bot Stud. 2013 Aug 21;54:12. doi: 10.1186/1999-3110-54-12 (PMC5432754; doi:10.1186/1999-3110-54-12)
Supplement: Supplementary file 1 — Additional file 1: Table S1: The primer pairs used in this study. (DOCX 18 KB) [file 40529_2012_16_MOESM1_ESM.docx]

Table S1. The primer pairs used in this study.

| Primers | Sequences | Used for |
| --- | --- | --- |
| BAmOsMADS45 | 5’-TAGGATCCGGATGGGGAGGGGTCGGGTG-3’  5’-TTGGATCCTCATGGTAGCCATGGGGG-3’ | Construction |
| Hd1-1 | 5’-CAAAATTCCACAAGAGCCATGCGA-3’  5’-GTCATGGAGGCGGCCTGGTCT-3’ | Sequencing |
| Hd1-2 | 5’-TTCTCCTCTCCAAAGATTCC-3’  5’-ACT GAG CAA GTA CCA ACA ATA T-3’ | Sequencing |
| Ehd1-1 | 5’-CTGAGCAACATTTGGCAGCA-3’  5’- GCACTCTGAGCCACTTGAGA-3’ | Sequencing |
| GUS | 5’-CCGTCCTGTAGAAACCCCA-3’  5’-AGTTCAGTTCGTTGTTCACACA-3 | Southern blotting |
| Act1 | 5’-ATGCTATCCCTCGTCTCGAC-3’  5’-TAGAAGCATTTCCTGTGCA-3’ | RT-PCR |
| Ehd1 | 5’-CTGAGCAACATTTGGCAGCA-3’  5’-GCACTCTGAGCCACTTGAGA-3’ | RT-PCR |
| Ehd2 | 5’-AAGAGGGTGTACGTGTGCCCGGA-3’  5’-AGCAGCAAGAAGCCTTGCACTCTC-3’ | RT-PCR |
| Hd1 | 5’-AGACCAGGCCGCCTCCATGAC-3’  5’-AAACGGCCCTTGATCCGGGGT-3’ | RT-PCR |
| Hd3a | 5’-TCACCTATGGCTCCAAGACC-3’  5’-CTAGGGGTAGACCCTCCTGC-3’ | RT-PCR |
| OsGI | 5’-TGGAGAAAGGTTGTGGATGC-3’  5’-GATAGACGGCACTTCAGCAGAT-3’ | RT-PCR |
| OsMADS45 | 5’-GGATGGGGAGGGGTCGGGTG -3’  5’-GCTGCCCGCGAACATGGTTG -3’ | RT-PCR |
| OsMADS14 | 5’-GAGTCCATTAACGAGCTTCAACG-3’  5’-GGCGAAAGGATAGAGGATGTAG-3’ | RT-PCR |
| OsMADS18 | 5’-CTCCGTGCTCTGTGACGCCG-3’  5’-ACAGATGGCCTTCATGTGTGACTTG-3’ | RT-PCR |
| OsMADS50 | 5’-GCCAGCGCCAGTACGCAGAA-3’  5’-GGAGCGACTTCTGCCAGGCA-3’ | RT-PCR |
| RFT1 | 5’-TCGTCCGGATCACTAACCTC-3’  5’-CCTTAGGGAGTATCTACACTGG-3’ | RT-PCR |
