# Supplementary figures and images for: Ectopic expression of OsMADS45 activates the upstream genes Hd3a and RFT1 at an early development stage causing early flowering in rice
Source: Bot Stud. 2013 Aug 21;54:12. doi: 10.1186/1999-3110-54-12 (PMC5432754; doi:10.1186/1999-3110-54-12)

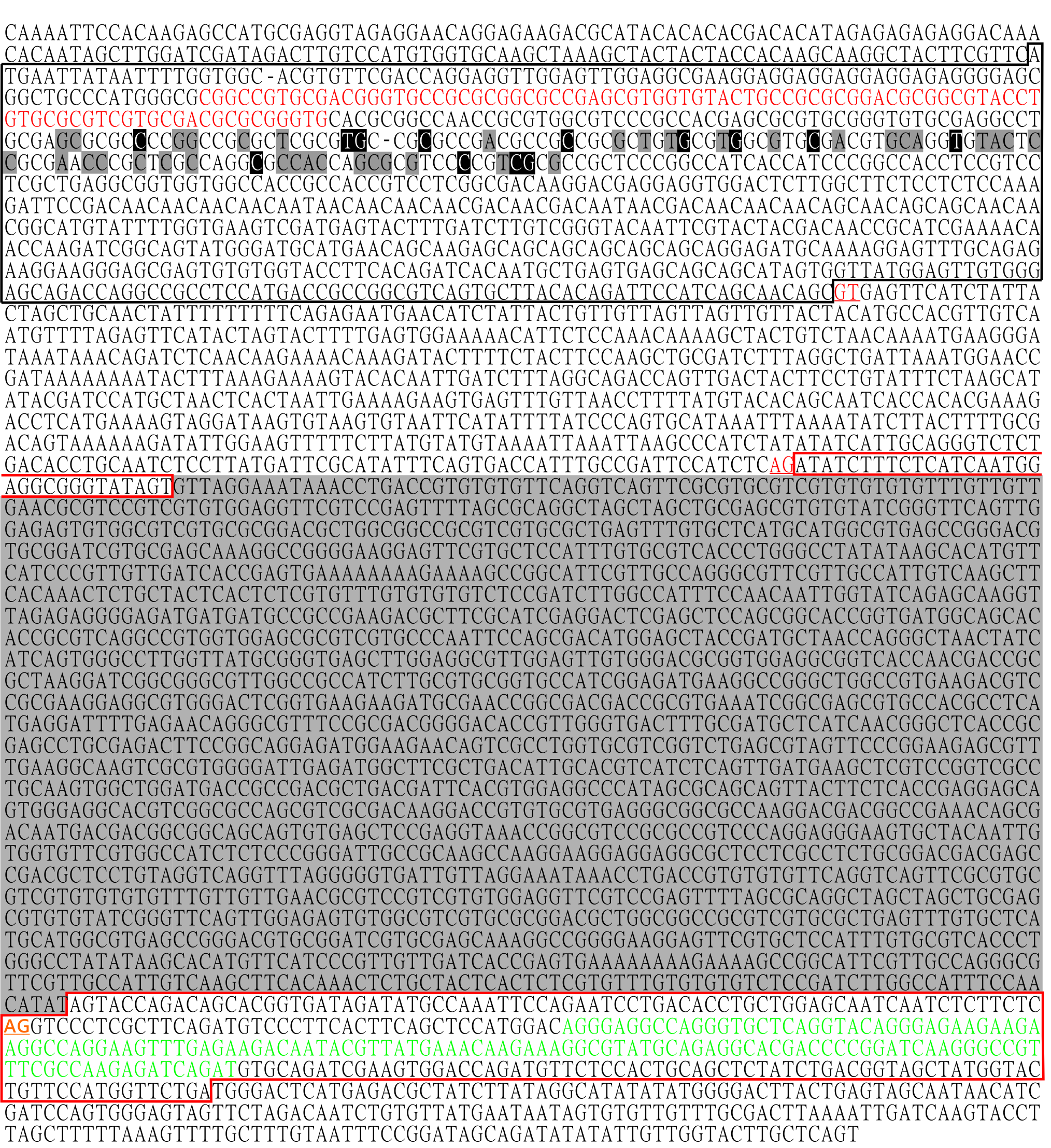

Supplement: Supplementary file 2 — Additional file 2: Figure S1: The genomic DNA sequence of the Hd1 gene of TNG67 rice. Two Hd1 exons are marked with boxes, and the intron junctions GT… AG are underlined and marked red. A total of 52 in/del and mismatched nucleotides in exon 1 (black boxed) compare with Nipponbare rice are marked with black (different bases) or grey shadings (additional bases in TNG67) or a dashed line to indicate the deleted bases. The red bases of exon 1 represent the zinc finger domain. A 1912-bp insertion is shaded grey within exon2 (red box), and the green bases represent the CCT domain. The proposed alternative splice site in exon 2 is underlined, in bold text and marked in orange. (TIF 18 MB) [file 40529_2012_16_MOESM2_ESM.tif]

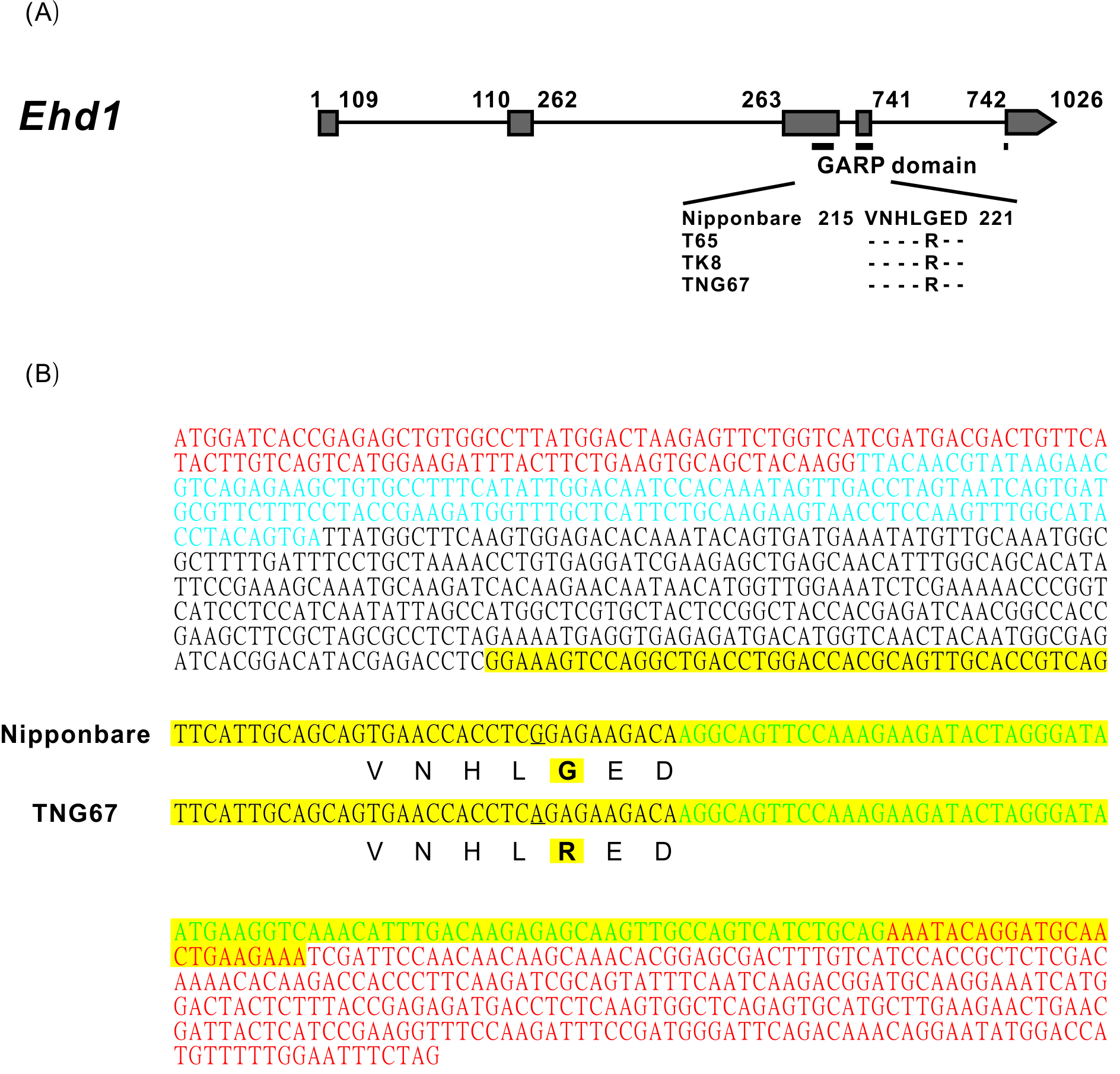

Supplement: Supplementary file 3 — Additional file 3: Figure S2: A schematic diagram showing the location of sequence variation and the detailed cDNA sequences of Ehd1 of TNG67 rice. (A) The functional glycine (G) in Nipponbare rice replaced by a non-functional arginine (R) in the 219th amino acid of Ehd1 as observed with T65 (Doi et al. 2004) and TK8 plants (Lin et al. 2011) is shown. (B) The cDNA sequences of Ehd1 of TNG67 rice with the replaced DNA (underlined) and amino acid sequences (G to R). Five exons are marked with different colors, and the GARP domain is highlighted in yellow. (TIF 14 MB) [file 40529_2012_16_MOESM3_ESM.tif]

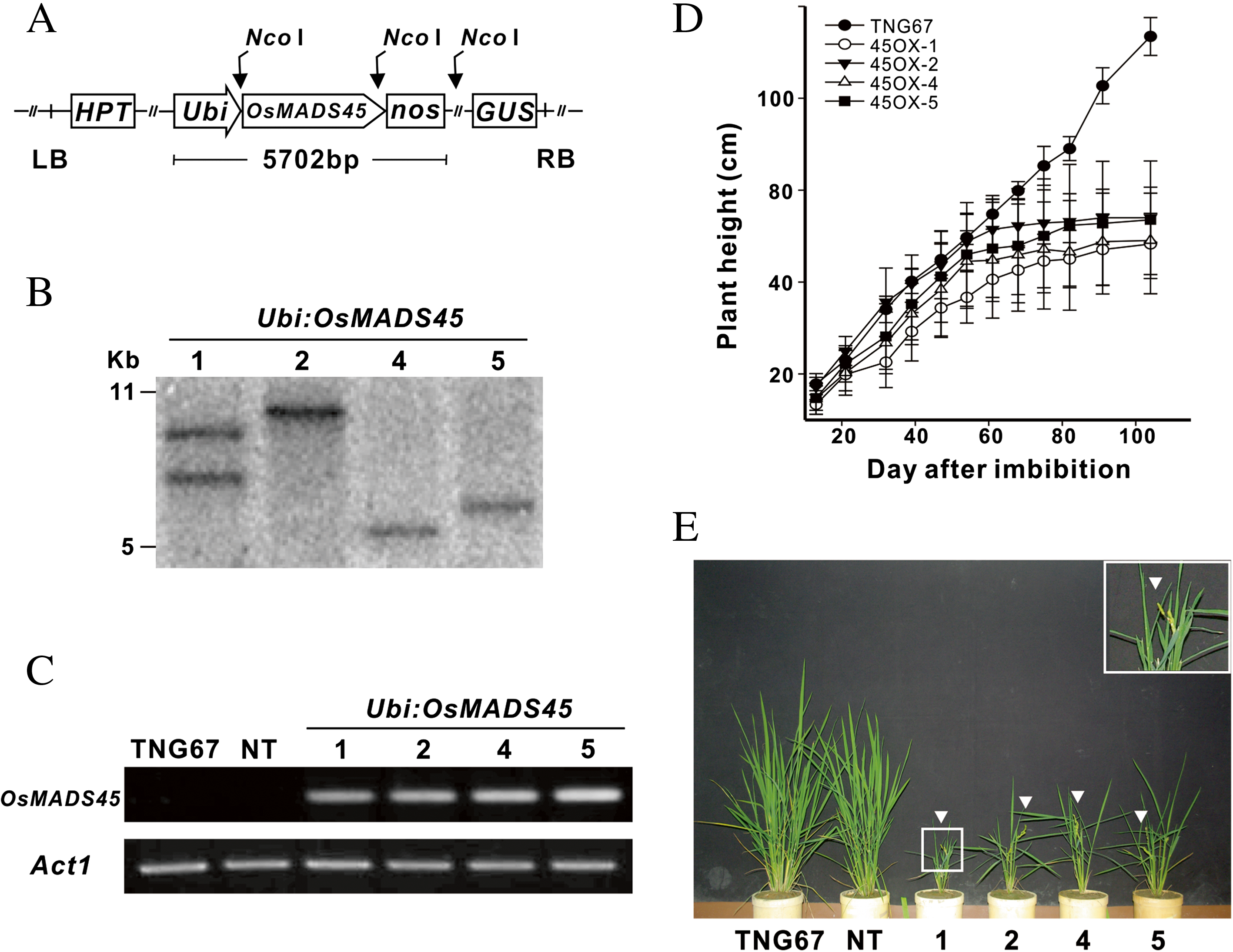

Supplement: Supplementary file 4 — Authors’ original file for figure 1 [file 40529_2012_16_MOESM4_ESM.tif]

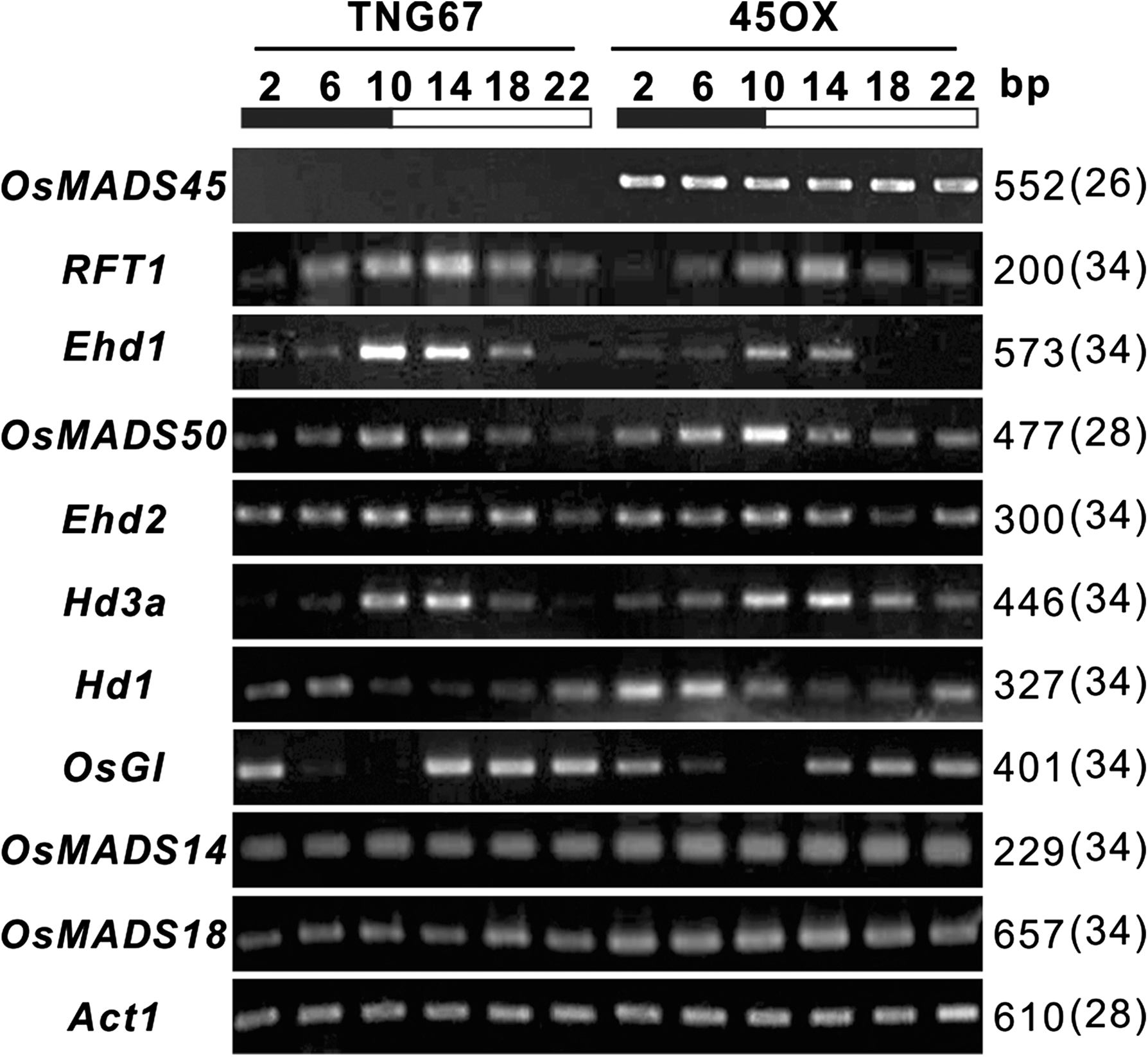

Supplement: Supplementary file 5 — Authors’ original file for figure 2 [file 40529_2012_16_MOESM5_ESM.tif]

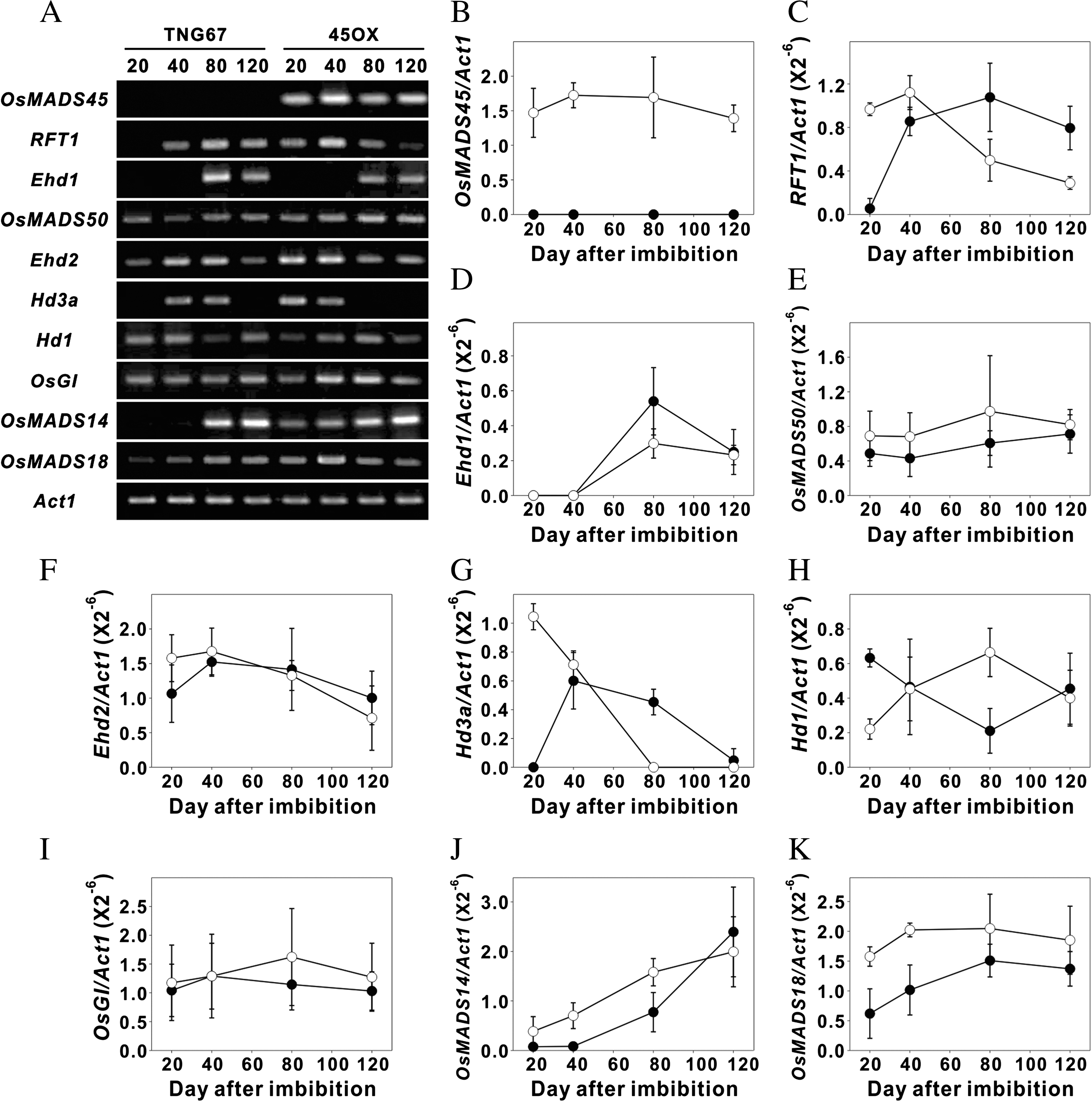

Supplement: Supplementary file 6 — Authors’ original file for figure 3 [file 40529_2012_16_MOESM6_ESM.tif]

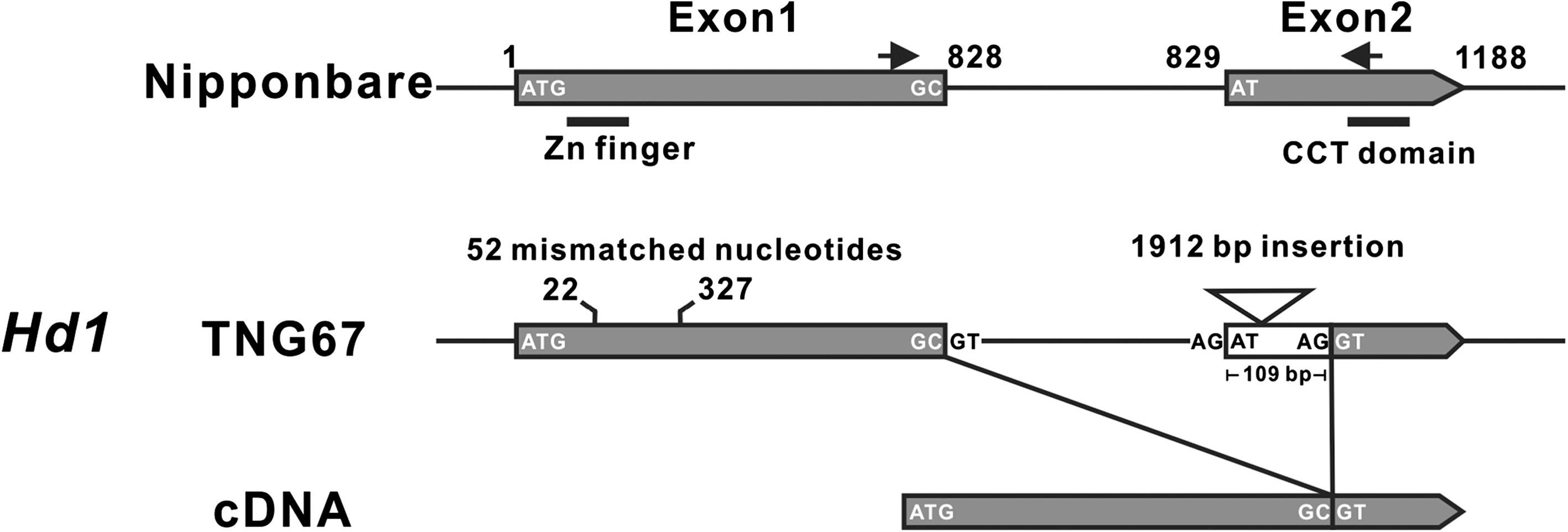

Supplement: Supplementary file 7 — Authors’ original file for figure 4 [file 40529_2012_16_MOESM7_ESM.tif]

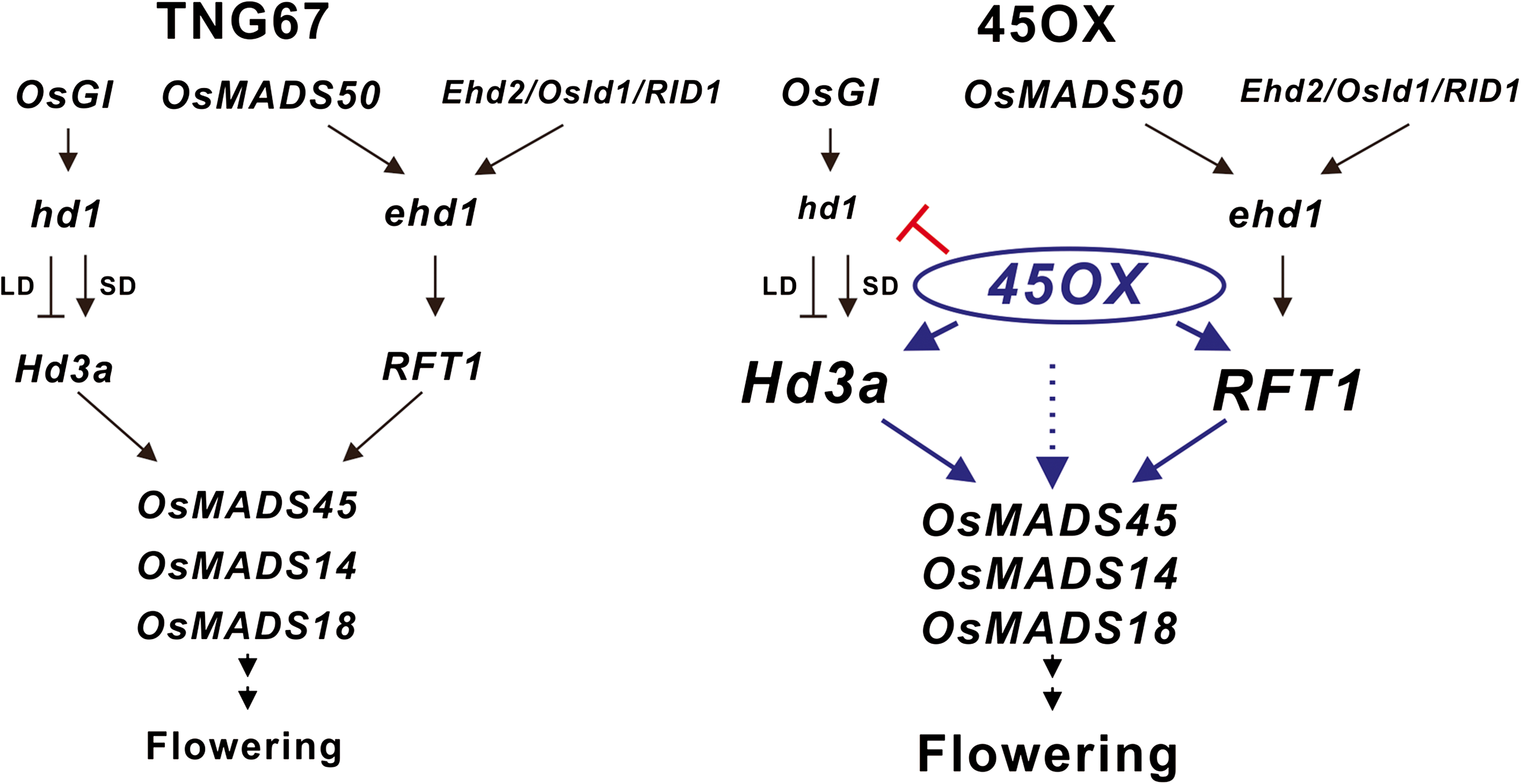

Supplement: Supplementary file 8 — Authors’ original file for figure 5 [file 40529_2012_16_MOESM8_ESM.tif]
